# Overview of dengue outbreaks in the southwestern Indian Ocean and analysis of factors involved in the shift toward endemicity in Reunion Island: A systematic review

**DOI:** 10.1371/journal.pntd.0010547

**Published:** 2022-07-28

**Authors:** Sarah Hafsia, Marion Haramboure, David Arthur Wilkinson, Thierry Baldet, Luce Yemadje-Menudier, Muriel Vincent, Annelise Tran, Célestine Atyame, Patrick Mavingui

**Affiliations:** 1 UMR PIMIT (Processus Infectieux en Milieu Insulaire Tropical) CNRS 9192-INSERM 1187-IRD 249-Université de La Réunion, île de La Réunion, France; 2 CIRAD, UMR TETIS, Sainte-Clotilde, île de La Réunion, France; 3 TETIS, Univ Montpellier, AgroParisTech, CIRAD, CNRS, INRAE, Sainte-Clotilde, île de La Réunion, France; 4 CIRAD, UMR ASTRE, Sainte-Clotilde, île de La Réunion, France; 5 ASTRE, Université Montpellier, CIRAD, INRAE, Sainte-Clotilde, île de La Réunion, France; 6 Santé publique France, Saint Denis, La Réunion, France; University of Calgary, CANADA

## Abstract

**Background:**

Dengue is the world’s most prevalent mosquito-borne viral disease. It is endemic in many tropical and subtropical countries and represents a significant global health burden. The first reports of dengue virus (DENV) circulation in the South West Indian Ocean (SWIO) islands date back to the early 1940s; however, an increase in DENV circulation has been reported in the SWIO in recent years. The aim of this review is to trace the history of DENV in the SWIO islands using available records from the Comoros, Madagascar, Mauritius, Mayotte, Seychelles, and Reunion. We focus in particular on the most extensive data from Reunion Island, highlighting factors that may explain the observed increasing incidence, and the potential shift from one-off outbreaks to endemic dengue transmission.

**Methods:**

Following the PRISMA guidelines, the literature review focused queried different databases using the keywords “dengue” or “*Aedes albopictus*” combined with each of the following SWIO islands the Comoros, Madagascar, Mauritius, Mayotte, Seychelles, and Reunion. We also compiled case report data for dengue in Mayotte and Reunion in collaboration with the regional public health agencies in these French territories. References and data were discarded when original sources were not identified. We examined reports of climatic, anthropogenic, and mosquito-related factors that may influence the maintenance of dengue transmission independently of case importation linked to travel.

**Findings and conclusions:**

The first report of dengue circulation in the SWIO was documented in 1943 in the Comoros. Then not until an outbreak in 1976 to 1977 that affected approximately 80% of the population of the Seychelles. DENV was also reported in 1977 to 1978 in Reunion with an estimate of nearly 30% of the population infected. In the following 40-year period, DENV circulation was qualified as interepidemic with sporadic cases. However, in recent years, the region has experienced uninterrupted DENV transmission at elevated incidence. Since 2017, Reunion witnessed the cocirculation of 3 serotypes (DENV-1, DENV-2 and DENV-3) and an increased number of cases with severe forms and deaths. Reinforced molecular and serological identification of DENV serotypes and genotypes circulating in the SWIO as well as vector control strategies is necessary to protect exposed human populations and limit the spread of dengue.

## Introduction

Dengue is the most widespread arboviral infection in the world, with half of the world’s population living in at-risk areas [1,2]. It largely spreads in tropical and subtropical regions, affecting at least 128 different countries [1–3]. Although the majority of about 390 million dengue cases estimated worldwide each year are asymptomatic, about 96 million report symptoms [3]. The disease can range from dengue fevers (DFs), with mild symptoms, to much more severe forms called dengue hemorrhagic fever [4]. Each year, an estimated 20,000 deaths are causally linked to dengue infections [5,6].

Dengue virus (DENV) has a positive-sense single-stranded linear RNA genome (11 kilobases). It belongs to the genus *Flavivirus*, which includes other mosquito-borne RNA viruses of medical importance, such as Yellow Fever, West Nile, Japanese encephalitis and Zika. Historically, dengue is thought to have emerged more than a thousand years ago [7,8]. The first isolations of DENV were achieved in the 1940s [9–11]. From 1948 to 1956, serological characterization identified the existence of 4 different DENV serotypes, DENV-1 to DENV-4 [7,8,11,12]. More recently, molecular analyses have highlighted the diversity of DENV genotypes belonging to each of the serotypes [7,13,14].

DENV is transmitted to humans by mosquito vectors, mainly *Aedes aegypti* and *Aedes albopictus* [15, 16]. Long-term immunity to a single infecting serotype can last for several decades [17]. Cross-immunity between heterologous serotypes can also exist, but does not prevent sympatric cocirculation of multiple serotypes during outbreaks [7,13,14]. Previous DENV infection may lead to more severe disease upon infection by a secondary serotype (i.e., secondary infection), through “antibody-dependent enhancement” (ADE), one of the main causes of severe and lethal DENV infections [18,19].

Observations of DF have been increasing since the end of the World War II [20]. Models predict that populations located in at-risk areas will increase from 3.5 billion today to 6.1 billion by 2080, the majority constrained to the tropical and subtropical belt [2]. The areas where DENV is only epidemic could become endemic or hyperendemic, and prevalence in areas where DENV is already endemic or hyperendemic is expected to increase significantly [2]. According to the Centers for Disease Control and Prevention, epidemic defines a sudden increase in cases of a disease above the expected norm in a given area, endemic refers to a disease with a constant or usual prevalence in a given area, and hyperendemic describes a disease with a persistent high prevalence in a given endemic area [21]. However, for DENV, “hyperendemic” can also refer to endemic circulation of multiple DENV serotypes [7,8].

The intensification of DENV circulation is associated with parameters that are strongly marked in the tropical and subtropical areas, namely (i) human population growth and associated substandard housing and unplanned urbanization; (ii) high vector population densities and lack of effective vector control methods; (iii) scarcity of resources for DENV prevention, surveillance, and treatment; and (iv) intensification of maritime and air traffic facilitating the import/export of vectors or DENV cases [22]. Due to their reduced connectivity and smaller size, island ecosystems, such as those of the South West Indian Ocean (SWIO), provide ideal settings for the study of factors that influence DENV circulation [23].

**Fig 1:**
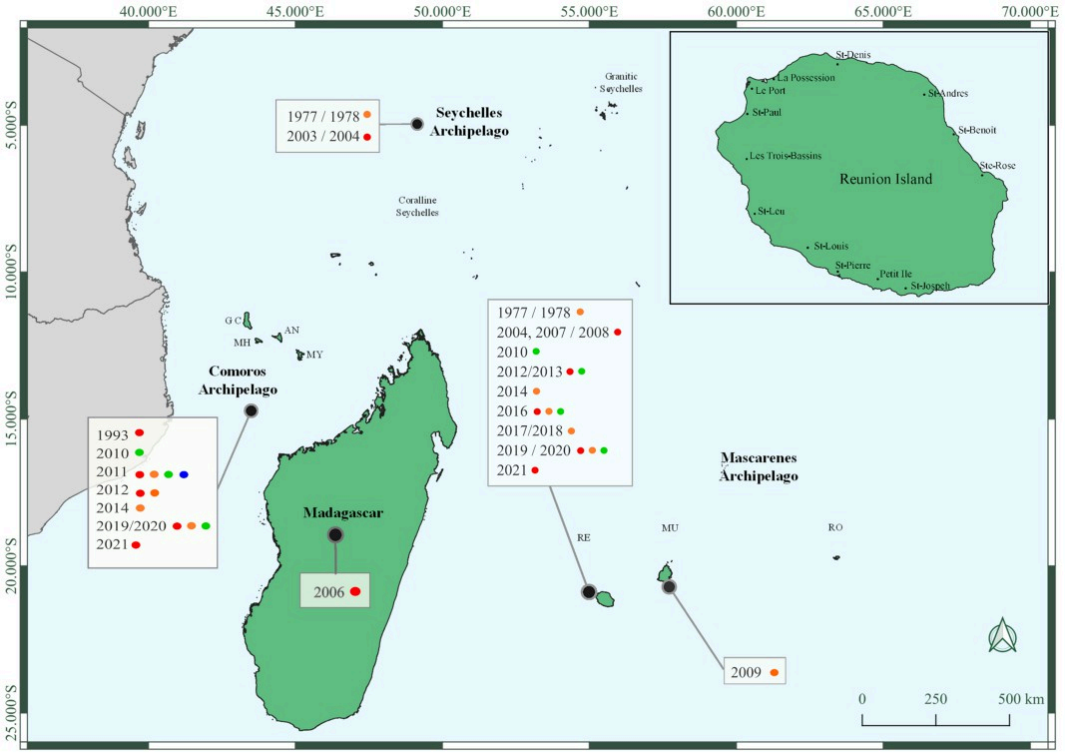
Islands of the southwestern Indian Ocean. The enlargement on the top right details the map of Reunion Island with the locations of the main cities. The rectangles show the dengue history in the region. The different dates correspond to the DENV outbreaks, and the colored circles represent the serotypes responsible for these outbreaks, red for DENV-1, orange for DENV-2, green for DENV-3, and purple for DENV-4. Cartographic data: administrative boundaries for Mayotte and Reunion Island come from OpenStreetMap; administrative boundaries for other countries come from ICPAC Geoportal. Software: QGIS3.10. Map was created using QGIS. Basemap: GADM data (https://www.gadm.org/data.html). AN, Anjouan; CS, Coralline Seychelles; DENV, dengue virus; GADM, Global Administrative Areas; GC, Grande Comore; GS, Granitic Seychelles; MH, Mohéli; MU, Mauritius; MY, Mayotte; RE, Reunion Island; RO, Rodrigues.

The SWIO islands include Madagascar, and the archipelagos of the Mascarenes (with Reunion, Mauritius, and Rodrigues), the Comoros (with Grande Comore, Mohéli, Anjouan, and Mayotte), the Seychelles (Fig 1), as well as numerous uninhabited islets known as the Iles Eparses (Europa, Juan de Nova, Glorieuses, and Tromelin). All inhabited SWIO islands have experienced sporadic dengue epidemics and are colonized by *Ae*. *aegypti* and/or *Ae*. *albopictus* vectors, with tropical climates favorable to their abundance [24]. Unlike many SWIO islands, Reunion, a French overseas territory, has a well-developed infrastructure providing healthcare services that allow for thorough surveillance of dengue cases as well as significant resources for vector control [25].

This review proposes to (i) retrace the history of DENV in the SWIO islands (the Comoros, Madagascar, Mauritius, Mayotte, Seychelles, and Reunion); (ii) decipher factors involved in the occurrence of DENV outbreaks in recent years; and (iii) summarize the control measures implemented. We explore whether DENV transmission is evolving toward endemicity, particularly in Reunion where epidemiological data are relatively abundant.

## Methods

The literature review applied the PRISMA (preferred reporting items for systematic reviews and meta-analyses) guidelines [26]. We used published data on DENV by searching in the PubMed and Google Scholar databases using the keywords “dengue” or “*Aedes albopictus*” simultaneously with each of the SWIO islands the Comoros, Madagascar, Mauritius, Mayotte, Seychelles, and Reunion. After sorting, each article was read in full. We also compiled an extensive database by extracting information from French dengue case reports in Reunion and Mayotte since 2007. These data were provided by the regional unit of the French national public health agency (https://www.santepubliquefrance.fr/regions/ocean-indien) in collaboration with the French Indian Ocean health agencies (ARS-OI and CIRE-OI). Only nonconfidential data records were used, and we did not have access to clinical data, impairing the use of guideline such as RECORD [27]. References reporting no original empirical data were disregarded. Overall, we examined 29 publications and 224 reports (168 from Reunion and 56 from Mayotte). We identified observational, interventional, and population-based records and extracted data to evaluate the number of serologically confirmed cases as well as severe forms and deaths. Missing fields as a result of broad data heterogeneity meant that multivariate meta-analyses were inappropriate. Thus, compiled data were used to examine trends in disease incidence and severity.

## Results

### Updating history of DENV circulation in the SWIO islands

The first dengue outbreak was documented in 1943 in the Comoros [28]. A dengue outbreak was reported in Reunion Island in 1977 to 1978 involving DENV-2 and *Ae*. *albopictus* as vector [29], with approximately 30% of the population infected [30]. Viral isolation from patient serum [31] also confirmed the spread of DENV-2 infection by *Ae*. *albopictus* in the Seychelles in 1976 to 1977, infecting nearly 80% of the population [32]. In 1993, DENV-1 outbreak occurred on the Comoros with *Ae*. *aegypti* as vector [33,34].

After a period described as “interepidemic” with sporadic cases, “microepidemics” occurred in the 2000s. First in the Seychelles, between 2003 and 2004, with more than 400 patients diagnosed and active transmission of DENV-1 confirmed by entomological studies [35]. Then in Reunion in 2004, 228 cases were diagnosed in the west of the island with DENV-1 and *Ae*. *albopictus* as the main vector [36]. The same DENV-1 serotype caused an outbreak in Toamasina, eastern Madagascar, 2 years later in 2006 [37]. DENV-1 was also implicated in small outbreaks in Reunion in 2007 and 2008 with 28 and 44 autochthonous cases, respectively (Fig 1). Occurrence of dengue-like syndromes was demonstrated by a sentinel surveillance system deployed in Madagascar between 2007 and 2008 [38]. *Ae*. *albopictus* was also suspected in the transmission of 234 DENV-2 cases in Mauritius in 2009 [39]. In 2010, autochthonous cases of DENV-3 were reported for the first time in the region, in the Comoros [40], Mayotte and Reunion (with 54 and 18 autochthonous cases, respectively) [41]. A 2011 seroepidemiological survey in the Comoros showed the circulation of different arboviruses, including the 4 DENV serotypes at high rates [42]. For example, as many as 80% of about 300 individuals had neutralizing antibodies to DENV on the island of Grande Comore [42]. In contrast, in 2008 a serological survey among 1,825 volunteer blood donors in Reunion revealed an overall seroprevalence of 3.1%, suggesting a population mostly naive for dengue infection [43]. In 2012, both DENV-1 and DENV-2 were implicated in an outbreak in Mayotte with 43 autochthonous cases, while in Reunion, DENV-1 and DENV-3 were diagnosed in 31 and 21 autochthonous cases in 2012 and in 2013, respectively. Then in 2014, only DENV-2 was reported in SWIO: The Comoros, Mayotte, and Reunion Island (with 470 and 29 autochthonous cases, respectively) [41]. Only 6 autochthonous cases were reported in Reunion 1 year later. In Mayotte, no dengue cases were reported between 2015 and 2018. In 2019, a new outbreak was observed in Mayotte with 186 confirmed cases related to DENV-1. This outbreak was uninterrupted and was followed by a highest transmission of DENV-1 in 2020 with 4,305 confirmed cases [41]. In addition, the first severe cases (including hemorrhage, hepatic injury, and vasoplegia) and 8 associated deaths were reported in Mayotte in 2020 [41]. No autochthonous cases of dengue have been reported since the end of 2020 in Mayotte [41].

Following the microepidemics described in Reunion between 2004 and 2015, a surveillance system was set up by the CIRE-OI (cellule interrégionale d’épidémiologie Océan Indien) that allowed more efficient monitoring of DENV outbreaks and the identification of DENV variants circulating on the island. These data (number of cases, severity and DENV serotypes) highlight a shift of epidemiological profile of dengue in Reunion from 2016, as detailed below.

### Epidemiology of dengue in Reunion Island

#### Number of confirmed cases and incidence rate of dengue

**Fig 2:**
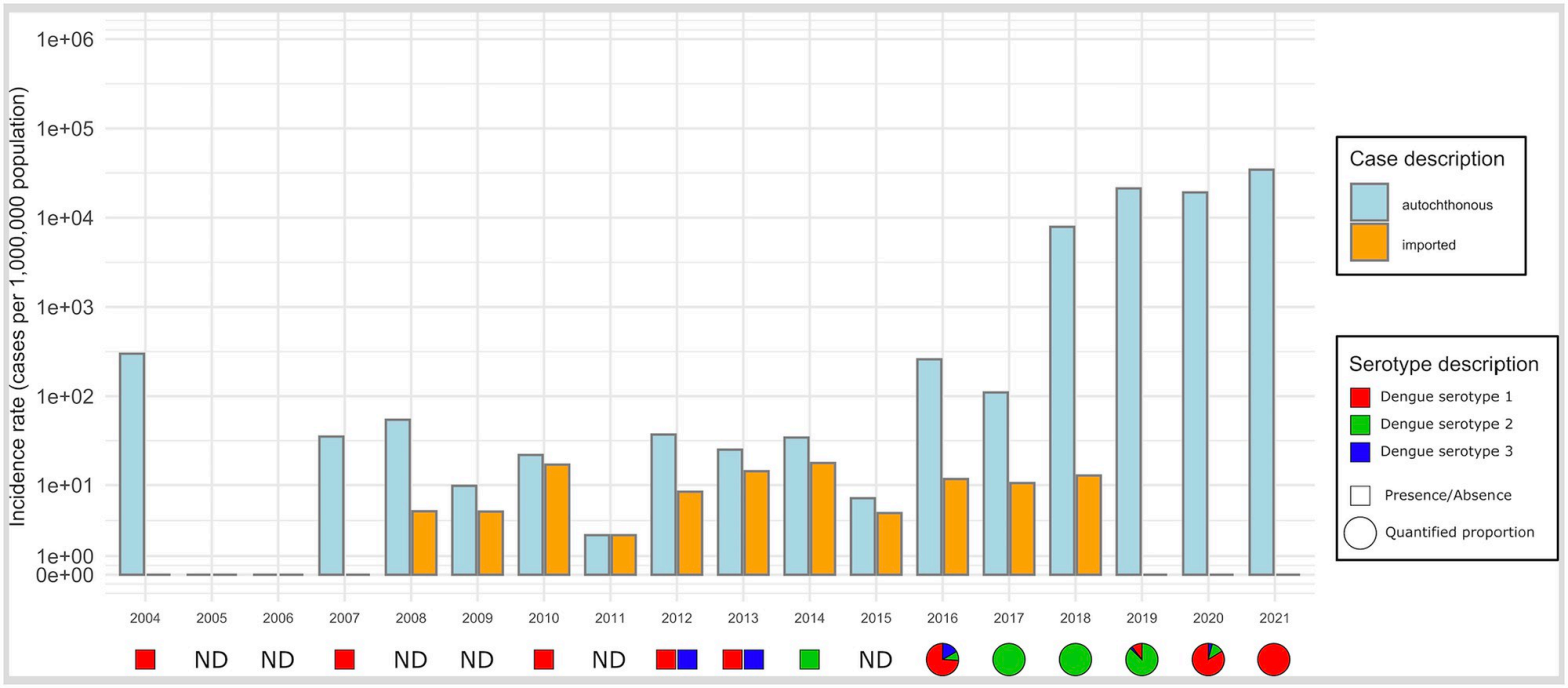
Incidence of autochthonous dengue cases per million population in Reunion from 2004 to 2021. Incidence is expressed in cases per million population for visualization purposes. The distribution of DENV serotypes are given for autochthonous cases based on the number of samples serotyped in 2016 (93 out of 221), 2017 (94 out of 94), 2018 (950 out of 6,770), 2019 (883 out of 18,217), 2020 (836 out of 16,414), and 2021 (978 out of 29,577). DENV, dengue virus; ND, unavailable data.

Following low-level DENV circulation from 2004 to 2015, a considerable increase in the annual number of dengue cases was reported from 2016. In 2016, the number of cases was 231 (221 autochthonous cases and 10 imported cases) with an incidence rate of 26 autochthonous cases per 100,000 people (Fig 2). A slight decrease in the annual number of cases was observed in 2017 with 103 dengue cases (94 autochthonous and 9 imported cases) and an incidence rate of 11 autochthonous cases per 100,000 people. During the period 2018 to 2021, the total annual number of autochthonous dengue cases increased significantly: 6,759 in 2018, 18,217 in 2019, 16,414 in 2020, and 29,577 in 2021. The population incidence was 790, 2,127, 1,916, and 3,448 autochthonous cases per 100,000 population in 2018, 2019, 2020, and 2021, respectively.

#### DENV serotypes circulating in Reunion Island

The 4 DENV serotypes have been detected in Reunion since 1977 to 1978: the 3 serotypes DENV-1, DENV-2 and DENV-3 in autochthonous cases and the 4 serotypes DENV-1, DENV-2, DENV-3, and DENV-4 in imported cases (Table 1).

**Table 1:** Summary of the number of confirmed cases of dengue and severe dengue disease in Reunion from 1977 to 2021.

Year	Total number of cases	Autochtonous cases	Imported cases	Dengue severity			DENV serotypes
				Hospitalizations	Severe cases	Deaths	
1977 to 1978	ND	ND	ND	ND	ND	ND	2^a^
2004	228	228	ND	ND	ND	ND	1^a^
2005	ND	ND	ND	ND	ND	ND	ND
2006	ND	ND	ND	ND	ND	ND	ND
2007	28	28	ND	7	0	0	1^a^
2008	48	44	4	ND	ND	ND	1^a^, 3^i^
2009	12	8	4	ND	ND	ND	2^i^
2010	32	18	14	1	0	0	1^i^, 3^a^^+^^i^, 4^i^
2011	4	2	2	6	0	0	ND
2012	38	31	7	0	0	0	1^a^, 3^a^
2013	33	21	12	4	0	0	1^a^^+^^i^, 3^a^^+^^i^
2014	44	29	15	0	0	0	2^a^^+^^i^
2015	10	6	4	0	0	0	ND
2016	231	221	10	18	3	0	1^a^, 2^a^, 3^a^
2017	103	94	9	14	0	0	1^i^, 2^a^^+^^i^, 4^i^
2018	6,770	6,759	11	154	27	6	2^a^
2019	18,217	18,217	ND	620	75	14	1^a^, 2^a^, 3^a^
2020	16,414	16,414	ND	787	108	22	1^a^^+^^i^, 2^a^^+^^i^, 3^a^
2021*	29,577	29,577	ND	1,111	245	33	1^a^

When serotyping was performed, the origin (i.e., from autochthonous or imported cases) is given.

^a^Serotype identified in autochthonous cases.

^i^Serotype identified in imported cases.

*In 2021, data available only from January to the beginning of October.

ND, unavailable data.

For autochthonous cases, DENV-1 was the only serotype circulating on the island in 2004, 2007, 2008, and 2021. Only DENV-2 circulated in 2009, 2014, 2017 and 2018 (Fig 2). DENV-3 was first detected on the island in 2010 and was the only serotype detected that year. After this first detection, DENV-3 cocirculated with DENV-1 in 2012 and 2013 and the cocirculation of the 3 serotypes DENV-1, DENV-2, and DENV-3 was reported in 2015 to 2016, 2019, and 2020 [41,44].

Data on DENV serotype distributions were often partial or incomplete; we thus limited our interpretation of serotype abundances in autochthonous cases to samples tested between 2017 and 2021 (Table 1). During this period, DENV-1 and DENV-2 were predominant, and a small number of coinfections were detected in 2019 and 2020 (Fig 2). In 2021, only DENV-1 was reported between January and August.

#### Dengue severity in Reunion Island

As observed in other countries, the clinical signs described in most reported dengue cases in Reunion are classic DF symptoms. Data regarding the severity of dengue combine reported hospitalizations due to symptoms such as respiratory distress, thrombopenia, lethargy, renal and hepatic failure, and/or more severe symptoms such as dengue shock syndrome (DSS), as well as a small number of cases of dengue hemorrhagic fever (DHF) and death. The first severe case of dengue was described in 2016 and the first dengue-related death in 2018. This recent severity of dengue in Reunion may be related with a significant increase in the number of cases and hospitalizations coinciding with the cocirculation of 3 DENV serotypes (DENV-1, DENV-2, and DENV-3) since 2016 (Table 1, Fig 2).

Strikingly, several severe ophthalmological cases related to DENV-1 infection have been reported, notably in 2020 and 2021 [41]. From 2019 to and 2021, secondary infections (individual who already had DF in the past) were reported in several patients, accounting for 6.7% of confirmed cases in 2020. Deaths in patients with secondary infections increased from 0 in 2018 to 7% in 2019, 18% in 2020, and 24% in 2021. The median age of dengue-infected patients is about 40. Severe forms and deaths mostly occurred in older patients with comorbidities such as diabetes and hypertension. However, increasing numbers of dengue-induced mortalities have been observed in younger patients (including children) without comorbidities [41].

#### Trends of transmission seasonality and the geographic distribution of dengue cases in Reunion Island from 2017 to 2021

Strong seasonal patterns in dengue incidence were observed in Reunion from 2017 to 2021. Outbreak peaks were observed between March and June, corresponding to the end of the austral summer, which is typically hot and humid, and then gradually decreases from June to July during the austral winter. From 2017 onward, a low level of DENV circulation was observed until the end of the year, unlike previous years when transmission stopped during the austral winter. This persistence of DENV during the interepidemic period is a new trend that may favor a rapid increase in cases early in the year.

The geographic distribution of dengue cases during the 2016 to 2020 outbreaks revealed a contrasting pattern in Reunion. Generally, the southern and western parts of the island are the most affected, while dengue incidence is generally low in the eastern and central parts. In 2016, the first cases were reported in Saint Joseph in the south of the island, then the dengue wave spread to the west and the north. In 2017, 2018 and 2019, most dengue cases occurred in the west, notably in the cities of Saint Paul, Saint Louis, and La Possession, and in the south especially in Saint Pierre and Petite-Île. Unlike previous years, the 2020 dengue outbreak was characterized by the circulation of DENVs in the west of the island at the beginning of the outbreak. Thereafter, the number of cases increased rapidly and a wider geographic spread was observed. In 2020, most DENV-3 cases were reported in the east, in particular in Saint André, while overall DENV circulation in this city was low. For 2021, the west of the island is the most affected, particularly in the cities of Le Port, La Possession, and Saint Paul. Several factors related to DENV genotypes, human populations, mosquito vectors, and environmental and weather, as well as the interactions between these factors, may explain the observed spatiotemporal distribution of dengue cases in Reunion over the past few years. We have also examined how some of these factors may explain the epidemiology of dengue in Reunion.

### Factors affecting the emergence and spread of DENV in Reunion Island

#### Human demographics

Reunion Island covers an area of 2,512 km^2^ for a population of about 850,000 inhabitants, with an average density of 339.8 inhabitants per km^2^. Between 2007 and 2012, the population increased by an average of 1% per year and since 2012, the population increased by 0.5% [45]. The population is expected to reach 1 million by 2030. Due to the mountainous nature of the center of the island, more than 50% of the population is located in coastal areas, where the density can reach over 1,000 inhabitants per km^2^ [46]. The main cities are Saint Denis in the north (147,931 inhabitants), Saint Paul in the west (104,519 inhab.), and Saint Pierre in the south (84,212 inhab.) (Fig 1).

From 2016 to 2021, a majority of dengue cases (approximately 95%) were observed in the western and southern coastal areas in dense population centers [43]. However, although equally densely populated, the northern region reported only a small percentage (approximately 3%) of cases, suggesting that demographic characteristics alone do not explain dengue transmission in Reunion. As far as we know, the relationship between demography and dengue circulation [47] has not been studied in Reunion.

#### Geographic mobility of human populations

Geographic mobility into and within Reunion contribute to the introduction and spread of DENV. The 2 airports (Roland Garros and Pierrefonds) handle over 2.5 million passengers a year, a number increasing on average by 3.5% per year since 2005. Most of Reunion’s air traffic is with Mauritius and mainland France. However, there are many direct and indirect air links with Southeast Asia, where dengue is endemic, thus facilitating the potential introduction of new dengue serotypes/genotypes to the island [41]. Indeed, from 2008 to 2020, between 1 and 9 confirmed dengue cases were imported each year from Asia (Cambodia, India, Indonesia, Malaysia, Myanmar, the Philippines, Sri Lanka, and Thailand), neighboring Indian Ocean islands (the Comoros archipelago, Madagascar, the Maldives, and the Seychelles), Oceania (French Polynesia), but also Central America (Nicaragua), South America (French Guiana and Brazil), the French Caribbean islands (Guadeloupe and Martinique), and Africa (Tanzania) [44]. Of note, in 2015 to 2016, 3 different serotypes (DENV-1, DENV-2, and DENV-3) were identified in 3 travelers returning from Indonesia and Malaysia [44], highlighting the diversity of DENV variants regularly introduced to the island. Also, comparison of the complete DENV-2 genomes from Reunion (2018) and Seychelles (2016) revealed 99.8% identity [48], suggesting that these DENV strains may have circulated between the SWIO islands.

**Fig 3:**
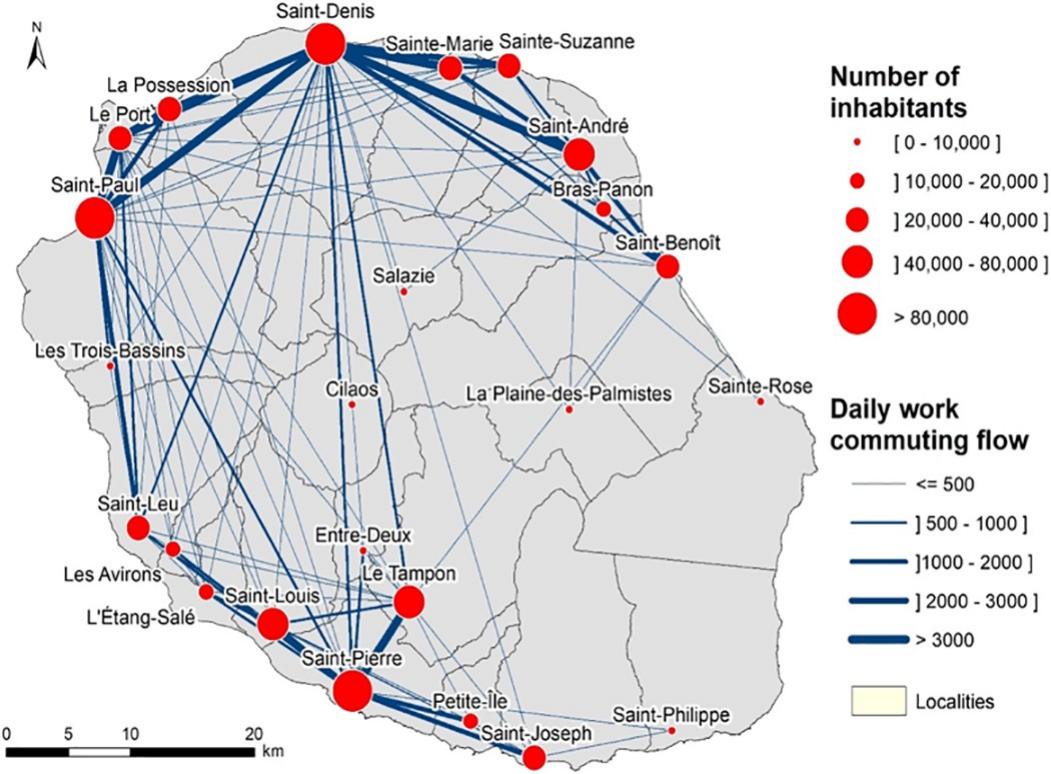
Population and daily commuting mobilities, Reunion Island, 2016. Population and daily commuting mobilities, Reunion Island, 2016. Census and mobility data are open data available from https://www.insee.fr/fr/statistiques; base map: French national geographic institute, BD TOPO—data available under the open license Etalab 2.0 (https://geoservices.ign.fr/bdtopo).

Within the island, population movements are continuous between all areas resulting in active daily interconnection (Fig 3). After a dengue introduction into a given area of the island, the circulation of the virus is progressive, spreading mainly locally around an epicenter through the vectors. Human population movement may contribute to the emergence of new epicenters, notably when outbreak spreads throughout the island, even in remote locations from the original epicenter.

#### Climatic factors

The climate of Reunion is subtropical, characterized by 2 seasons: the austral winter (or “dry season”) from May to October, with mild temperatures (average minima of 17 to 20°C and average maxima of 26 to 28°Ca, at sea level), less precipitation and trade winds blowing regularly from the east; the austral summer (or “rainy season”), from November to April, with higher temperatures (average minima ranging from 21 to 24°C, and average maxima from 28 to 31°C, at sea level), humidity and rainfall (http://www.meteofrance.re/climat/description-du-climat). Local variations in climate are pronounced due to the highly uneven relief and different wind exposures. The east is exposed to oceanic humidity brought by the trade winds and registers some world rainfall records, with cumulative measures exceeding 10 meters per year in some places (annual maximum of 15,931 mm), and even in the dry season, rainfall remains significant. Conversely, the western leeward side of the island benefits from the shelter of the relief and is much drier (annual minimum of 183 mm).

**Fig 4:**
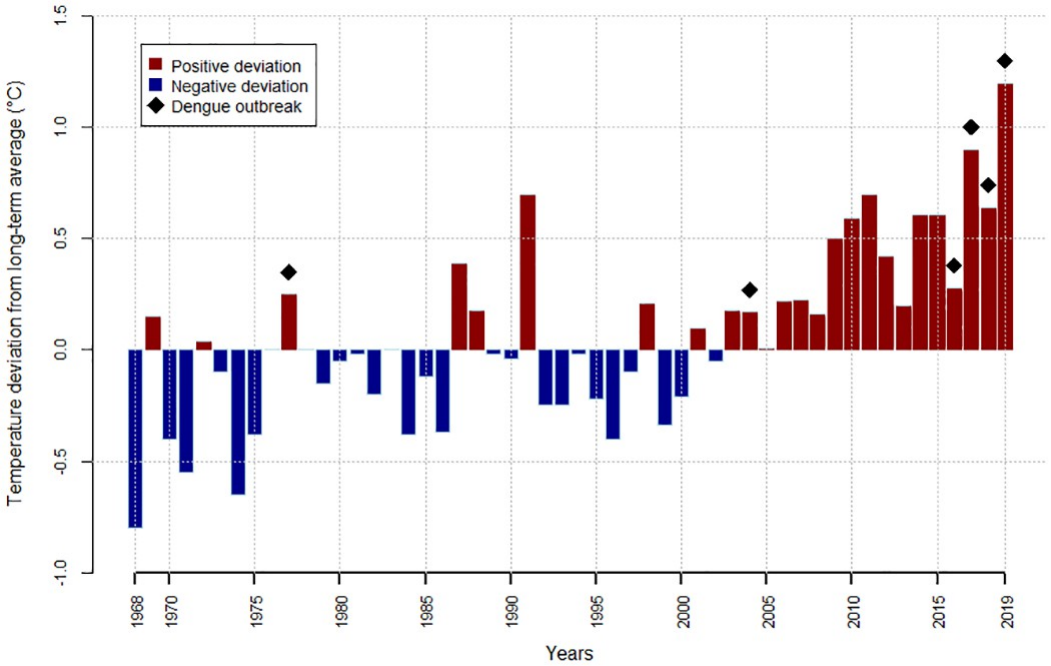
Average annual temperature in Reunion Island, 1968 to 2019 (deviation from long-term average). Black diamonds indicate dengue outbreaks.

Even if some contrasts exist, this subtropical climate is suitable for the development of the dengue vector, *Ae*. *albopictus*, throughout the year, particularly on the coastal parts of the island [24,49]. This annual persistence seems to be more marked in recent years with mosquito densities remaining relatively high on the coast during the austral winter. The current dengue outbreak is occurring under favorable conditions as records of temperatures have been reported since 2017: 2019 was the warmest year recorded since measurements began over 50 years ago (Fig 4). Indeed, warmer temperatures favor the development of vectors from aquatic to adult stages (see next section), as well as dengue transmission, by shortening the extrinsic incubation period and increasing vector competence [50]. Interestingly, the first 2 dengue outbreaks in Reunion (1977 and 2004) occurred during hot years with above-normal annual temperatures (Fig 4). The impact of rainfall on current dengue outbreaks is less clear. Indeed, 2017 was classified by Météo France as the third wettest year since 1972, whereas 2019 was the sixth driest year in 50 years.

#### Entomological and environmental factors

Twelve mosquito species including *Ae*. *albopictus* and *Ae*. *aegypti* are recorded in Reunion [51]. As in Madagascar, Mauritius and Seychelles, *Ae*. *albopictus* is the main dengue vector in Reunion [16]; only a few populations of *Ae*. *aegypti* persist in certain isolated areas [49]. *Ae*. *albopictus* is present throughout the island and remains active up to 1,200 m [24]. The abundance of *Ae*. *albopictus* is high during the austral summer, with a peak in March, although some variation is observed across the island [52]. Temperatures positively impact the development of aquatic stages [53]. Likewise, precipitation favors the creation of breeding sites, which are necessary for larval development [24]. However, heavy rainfall can sometimes wash out breeding sites, increasing mortality of aquatic stages [54].

Biotic factors, and in particular human behavior, also have an important effect on the population dynamics of *Ae*. *albopictus*. The storage of water in containers (e.g., flower plates or vases) provides breeding sites, which can be productive even during dry seasons [24]. These anthropogenic breeding sites favor the transmission and endemicity of dengue in tropical areas as observed for *Ae*. *aegypti* [55,56]. In Reunion, the monitoring of breeding sites conducted by the Regional Health Agency indicates that anthropogenic sites are more numerous in the western part of the island [52]. Indeed, the drier climate in this region obliges the inhabitants to store water. These reasons (higher temperature, and breeding site availability throughout the year), may partly explain the higher abundances of *Ae*. *albopictus* in the west of the island, and the higher DENV transmission.

### Control strategies against DENV in Reunion Island and SWIO islands

In the absence of specific treatments to control human infection by DENVs, the availability of vaccines would be the preferred method to reduce worldwide DENV transmission, both in local populations and travelers. However, a tetravalent vaccine with a high level of protection against all DENV serotypes is not yet available. Moreover, the Dengvaxia vaccine is currently subject to safeguards [57] and its use is not recommended for French overseas territories. Therefore, controlling *Ae*. *albopictus* populations, the main vector of DENV in the SWIO islands, is the only affordable measure to limit the transmission of DENV.

The current implementation of vector control against *Ae*. *albopictus* in Reunion and the other SWIO islands is mainly based on mechanical suppression of the breeding sites and chemical treatments with insecticides. In Reunion, since 2007, vector control programs are performed using *Bti* (*Bacillus thuringiensis* var. *israelensis* toxins) for larval control and deltamethrin (pyrethroids) against adults [58]. In Mauritius, in addition to deltamethrin-based adulticide treatments, temephos (organophosphate) is used against larvae. However, the effectiveness of vector control is limited by the difficulty of accessing private gardens that do not allow an exhaustive coverage of the areas to be treated [24] and by the multiplicity of cryptic breeding sites, making their treatment difficult. Besides, the control of mosquito populations using insecticides is increasingly threatened by the selection of resistance mechanisms and is disfavored due to the associated collateral environmental damage.

In recent years, several alternative, innovative and environmentally-friendly methods have been developed to control *Ae*. *albopictus* [59–61]. Among these methods, the sterile insect technique (SIT) is based on the principle of releasing males rendered sterile by irradiation, into the environment to mate with wild females [62,63]. These females will therefore not produce viable embryos, resulting in the population reduction. Pilot trials have been conducted in Italy to reduce *Ae*. *albopticus* populations [64,65], and a field trial is ongoing in Reunion [66]. In addition to SIT, incompatible insect technique (IIT) is also considered. IIT relies on the use of the maternally inherited Alphaproteobacteria *Wolbachia* to induce sterility in target populations through the cytoplasmic incompatibility (CI) phenotype. In mosquitoes, *Wolbachia* induce a form of embryonic death or CI resulting from sperm-egg incompatibility occurring when *Wolbachia* infected males mate with uninfected females or females infected with an incompatible *Wolbachia* strain [67]. IIT targeting *Ae*. *albopictus* has been successfully applied in a field trial in the United States [68]. In Reunion, IIT facilitated by the use of a selective sexing-strain [69] has shown promising results for controlling mosquito populations. WHO recommend integrated mosquito management to sustainably reduce their densities and arbovirus transmission [70]. Combining SIT and IIT technologies [71] has been shown to have an enhanced impact on *Aedes* control in China and Thailand [72,73].

## Discussion

This review provides an update on dengue outbreaks in the SWIO region and discusses factors that may explain the uninterrupted circulation of the virus since 2017 in Reunion leading to an endemic situation. Based on recent public health data, we showed that different dengue serotypes are currently circulating in the islands of the SWIO region where competent mosquito vectors are present. This is consistent with a more widespread trend observed across the tropical belt and even beyond in subtropical and temperate regions colonized by *Aedes* vectors [8,20]. Besides the morbidity and mortality induced by each serotype, data suggest that the successive or cocirculation of DENV-1, DENV-2, and DENV-3 serotypes in Reunion may have contributed to the increase in disease severity through the ADE effect [19]. Indeed, the epidemiological profile of DENV has significantly changed since 2017 in Reunion with an increased incidence of both mild and severe disease, including in young patients. In addition, populations in the SWIO islands, including Reunion, experience a high prevalence of preexisting chronic conditions such as diabetes and heart disease, making comorbidities in local DENV infection common, and thus representing aggravating factors for the development of severe forms and deaths [74]. Another striking change is the increasing appearance of DENV-1-related ophthalmic disorders since late 2020.

Despite a reinforced vector control program, the increased DENV incidence observed in Reunion these last 4 years is not an exception worldwide. It may be explained by several factors such as (i) the human population of Reunion has increased 1.6 times in less than 30 years, 36% of whom are under 20 years old, and most of them were naive to DENV; (ii) human behaviors such as mobility, use of water storage containers; (iii) environmental and climatic factors that show, respectively, a deterioration of natural environments and an increase in temperatures; and (iv) the abundant vector populations of *Ae*. *albopictus* that are maintained almost year-round, including during the winter period.

The DENV outbreak that started in 2017 persists until today, suggesting that DENV has become endemic in Reunion. Mayotte seems to follow the same shift as Reunion, as previous microepidemics without severe cases have been replaced by a continuous circulation since 2019, with low level circulation during interepidemic periods. Due to the increasing public health burden caused by DENV in the SWIO islands, as well as the shift in DENV-associated pathologies, it is clear that DENV should be categorized as a major public health priority in the region, requiring multilevel control and intervention measures. Because Reunion and Mayotte are French territories welcoming many international travelers each year, they can be considered as a potential sources of DENV introduction to Europe and the world. Increased importation of DENV into southern Europe where *Ae*. *albopictus* has recently established, could present a serious health risk to European populations.

### Limitations

We queried 2 bibliographic databases and cross-referenced the information with exhaustive data from health agencies. We cannot exclude the possibility that some articles may have escaped us, in particular concerning the dating of the first SWIO outbreaks, since the gray literature indicates earlier dates. The lack of access to detailed clinical data prevented the application of any protocol or meta-analysis. The lack of completeness of serology and its heterogeneity may have introduced a bias in our estimates of disease incidence.

## Conclusions

Despite the implementation of a reinforced surveillance of dengue as a compulsory disease and years of vector control program mainly based on insecticides, the DENV circulation that was sporadic is tending toward an endemic pattern in Reunion and probably in the SWIO region. Thus, emphasizing the fact that this health risk must be addressed through a holistic approach at the regional and global levels.

**Key Learning Points**

- Strong socioeconomic links exist between the South West Indian Ocean (SWIO) islands and Southeast Asia (especially China, Thailand, and India), the region of the world most affected by dengue, which may facilitate the regular introduction of dengue virus (DENV) serotypes/genotypes into the region.
- Climatic (temperature and rainfall) and anthropogenic (demography and human activities) factors in the west of Reunion favor the abundance of *Aedes*. *albopictus* populations and consequently the transmission of dengue.
- Certain indicators illustrate the endemic trend of dengue in Reunion: (i) uninterrupted circulation of DENV since 2017 in a mostly naive population; (ii) cocirculation of different serotypes; and (iii) an increase number of cases with severe forms and deaths.
- Conventional vector control actions (mechanical suppression of breeding sites and treatments with insecticides) must be reinforced by alternative methods as part of an integrated strategy. In this context, pilot trials of sterile insect technique (SIT) and incompatible insect technique (IIT) are underway against *Ae*. *albopictus* in Reunion.

**Top Five Papers**

1. Vasilakis N, Weaver SC. Chapter 1 The History and Evolution of Human Dengue Emergence. In: Advances in Virus Research [Internet]. Elsevier; 2008 [cited 2021 May 19]. p. 1–76. Available from: https://linkinghub.elsevier.com/retrieve/pii/S0065352708004016
2. Saeed O, Asif A. Dengue virus disease; the origins. In: Dengue Virus Disease [Internet]. Elsevier; 2020 [cited 2021 May 19]. p. 9–16. Available from: https://linkinghub.elsevier.com/retrieve/pii/B9780128182703000023
3. Messina JP, Brady OJ, Golding N, Kraemer MUG, Wint GRW, Ray SE, et al. The current and future global distribution and population at risk of dengue. Nat Microbiol. 2019 Sep;4(9):1508–15.
4. Delatte H, Gimonneau G, Triboire A, Fontenille D. Influence of Temperature on Immature Development, Survival, Longevity, Fecundity, and Gonotrophic Cycles of Aedes albopictus, Vector of Chikungunya and Dengue in the Indian Ocean. J Med Entomol. 2009 Jan 1;46(1):33–41.
5. Tran A, Mangeas M, Demarchi M, Roux E, Degenne P, Haramboure M, et al. Complementarity of empirical and process-based approaches to modelling mosquito population dynamics with *Aedes albopictus* as an example—Application to the development of an operational mapping tool of vector populations. PLoS ONE. 2020 Jan 17;15(1):e0227407.

## References

[1] Brady OJ, Gething PW, Bhatt S, Messina JP, Brownstein JS, Hoen AG, et al. Refining the Global Spatial Limits of Dengue Virus Transmission by Evidence-Based Consensus. PLoS Negl Trop Dis. 2012;6:e1760. doi: 10.1371/journal.pntd.0001760 22880140 PMC3413714

[2] Messina JP, Brady OJ, Golding N, Kraemer MUG, Wint GRW, Ray SE, et al. The current and future global distribution and population at risk of dengue. Nat Microbiol. 2019;4:1508–1515. doi: 10.1038/s41564-019-0476-8 31182801 PMC6784886

[3] Bhatt S, Gething PW, Brady OJ, Messina JP, Farlow AW, Moyes CL, et al. The global distribution and burden of dengue. Nature. 2013;496:504–507. doi: 10.1038/nature12060 23563266 PMC3651993

[4] World Health Organization. Dengue and severe dengue. World Health Organ 2021. https://www.who.int/news-room/fact-sheets/detail/dengue-and-severe-dengue (accessed July 8, 2021).

[5] Stanaway JD, Shepard DS, Undurraga EA, Halasa YA, Coffeng LE, Brady OJ, et al. The global burden of dengue: an analysis from the Global Burden of Disease Study 2013. Lancet Infect Dis. 2016;16:712–723. doi: 10.1016/S1473-3099(16)00026-8 26874619 PMC5012511

[6] About Dengue: What You Need to Know | Dengue | CDC 2019. https://www.cdc.gov/dengue/about/index.html (accessed July 8, 2021).

[7] Vasilakis N, Weaver SC. Chapter 1 The History and Evolution of Human Dengue Emergence. In: Maramorosch K, Shatkin AJ, Murphy FA, editors. Adv. Virus Res., vol. 72, Elsevier; 2008, p. 1–76. 19081488 10.1016/S0065-3527(08)00401-6

[8] Gubler DJ. Dengue/Dengue Haemorrhagic Fever: History and Current Status. In: Bock G, Goode J, editors. Novartis Found. Symp., Chichester, UK: John Wiley & Sons, Ltd; 2008, p. 3–22.

[9] Sabin AB, Schlesinger RW. Production of Immunity to Dengue with Virus Modified by Propagation in Mice. Science. 1945;101:640–642. doi: 10.1126/science.101.2634.640 17844088

[10] Sabin AB, Young I. A Complement Fixation Test for Dengue. Exp Biol Med. 1948;69:478–480. doi: 10.3181/00379727-69-16761p 18106662

[11] Hotta S. Experimental Studies on Dengue: I. Isolation, Identification and Modification of the Virus. Journal. Infect Dis. 1952;90:1–9. doi: 10.1093/infdis/90.1.1 Infectious Diseases, vol. 90, no. 1, Jan. 1952, pp. 1–9. 14888958

[12] Hammon WMD, Rudnick A, Sather GE. Viruses Associated with Epidemic Hemorrhagic Fevers of the Philippines and Thailand. Science. 1960;131:1102–1103. doi: 10.1126/science.131.3407.1102 14399343

[13] Weaver SC, Vasilakis N. Molecular evolution of dengue viruses: Contributions of phylogenetics to understanding the history and epidemiology of the preeminent arboviral disease. Infect Genet Evol. 2009;9:523–540. doi: 10.1016/j.meegid.2009.02.003 19460319 PMC3609037

[14] Chen R, Vasilakis N. Dengue—Quo tu et quo vadis? Viruses. 2011;3:1562–1608. doi: 10.3390/v3091562 21994796 PMC3187692

[15] Kramer MUG, Reiner RC Jr, Brady OJ, Messina JP, Gilbert M, et al. past and future spread of the arbovirus vectors Aedes aegypti and Aedes albopictus. Nat Microbiol. 2019;4:854–863. doi: 10.1038/s41564-019-0376-y 30833735 PMC6522366

[16] Paupy C, Delatte H, Bagny L, Corbel V, Fontenille D. Aedes albopictus, an arbovirus vector: From the darkness to the light. Microbes Infect. 2009;11:1177–1185. doi: 10.1016/j.micinf.2009.05.005 19450706

[17] Sabin AB. Research on Dengue during World War II. Am J Trop Med Hyg. 1952;1:30–50. doi: 10.4269/ajtmh.1952.1.30 14903434

[18] Guzman MG, Alvarez M, Halstead SB. Secondary infection as a risk factor for dengue hemorrhagic fever/dengue shock syndrome: an historical perspective and role of antibody-dependent enhancement of infection. Arch Virol. 2013;158:1445–1459. doi: 10.1007/s00705-013-1645-3 23471635

[19] Katzelnick LC, Gresh L, Halloran ME, Mercado JC, Kuan G, Gordon A, et al. Antibody-dependent enhancement of severe dengue disease in humans. Science. 2017;358:929–932. doi: 10.1126/science.aan6836 29097492 PMC5858873

[20] Brady OJ, Hay SI. The global expansion of dengue: how Aedes aegypti mosquitoes enabled the first pandemic arbovirus. Annu Rev Entomol. 2020;65:191–208. doi: 10.1146/annurev-ento-011019-024918 31594415

[21] CDC. Principles of Epidemiology. Lesson 1 Section 11, CDC 2012. https://www.cdc.gov/csels/dsepd/ss1978/lesson1/section11.html.

[22] Saeed O, Asif A. Dengue virus disease; the origins. In: Saeed O, Qureshi AI, editors. Dengue Virus Dis. Orig. Outbreak, Elsevier; 2020, p. 9–16.

[23] Tortosa P, Pascalis H, Guernier V, cardinale E, Le Corre M, Goodman SM, et al. deciphering arboviral emergence within insular ecosystems. Infect Genet Evol. 2012;12(1333–1339). doi: 10.1016/j.meegid.2012.03.024 22504353

[24] Delatte H, Dehecq JS, Thiria J, Domerg C, Paupy C, Fontenille D. Geographic Distribution and Developmental Sites of Aedes albopictus (Diptera: Culicidae) During a Chikungunya Epidemic Event. Vector-Borne Zoonotic Dis. 2008;8:25–34. doi: 10.1089/vbz.2007.0649 18171104

[25] Benkimoun S, Atyame C, Haramboure M, Degenne P, Thebault H, et al. Dynamic mapping of dengue basic reproduction number. Results in Physics. 2021;29(104687).

[26] Moher D, Liberati A, Tetzlaff J, Altma DA. The PRISMA Group. Preferred reporting items for systematic reviews and meta-analyses: the PRISMA statement. PLoS Med. 2009;6(e1000097). doi: 10.1371/journal.pmed.1000097 19621072 PMC2707599

[27] Benchimol EI, Smeeth L, Guttmann A, Harron K, Moher D, Petersen I, et al. The reporting of studies conducted using observational routinely-collected health data (RECORD) statement. PLoS Med. 2015;12:e1001885. doi: 10.1371/journal.pmed.1001885 26440803 PMC4595218

[28] McCarthy DD, Brent RH. An account of an outbreak of dengue in Dzaoudi. Comoro Islands. E. Afr Med J. 1943;20:293–298.

[29] Coulanges P, Clerc Y, Jousset FX, Rodhain F, Hannoun C. Dengue on Réunion. Isolation of a strain at the Pasteur Institute of Madagascar. Bull Soc Pathol Exot Filiales. 1979;72:205–209. 554772

[30] Kles V, Michault A, Rodhain F, Mevel F, Chastel C. A serological survey regarding Flaviviridae infections on the island of Réunion (1971–1989). Bull Soc Pathol Exot. 1994;87:71–76. 8061530

[31] Metselaar D, Grainger CR, Oei KG, Reynolds DG, Pudney M, Leake CJ, et al. An outbreak of type 2 dengue fever in the Seychelles, probably transmitted by Aedes albopictus (Skuse). Bull World Health Organ. 1980;58:937–943. 6971192 PMC2395999

[32] Calisher CH, Nuti M, Lazuick JS, Ferrari JDM, Kappus KD. Dengue in the Seychelles. Bull World Health Organ. 1981;59:619–622. 6976229 PMC2396093

[33] Boisier P, Morvan J, Laventure S, Charrier N, Martin E, Ouledi A, et al. Dengue 1 epidemic in the Grand Comoro Island (Federal Islamic Republic of the Comores). March-May 1993. Ann Soc Belg Med Trop. 1994;74:217–229. 7840689

[34] Ouledi A, Toyb M, Aubry P, Gaüzere B-A. Heath history and health challenges in the Union of The Comoros in 2012. Médecine Santé Trop. 2012;22:346–354. doi: 10.1684/mst.2013.0135 23485662

[35] D’Ortenzio E, Balleydier E, Baville M, Filleul L, Renault P. Dengue à la Réunion et dans les îles du sud-ouest de l’océan Indien. Médecine Mal Infect. 2011;41:475–479. doi: 10.1016/j.medmal.2010.11.021 21295427

[36] Pierre V, Thiria J, Rachou E, Sissoko D, Lassale C, Renault P. Epidémie de dengue 1 à la Réunion en 2004. 2005.

[37] Ratsitorahina M, Harisoa J, Ratovonjato J, Biacabe S, Reynes J-M, Zeller H, et al. Outbreak of Dengue and Chikungunya Fevers, Toamasina, Madagascar, 2006. Emerg Infect Dis. 2008;14:1135–1137. doi: 10.3201/eid1407.071521 18598641 PMC2600361

[38] Randrianasolo L, Raoelina Y, Ratsitorahina M, Ravolomanana L, Andriamandimby S, Heraud J-M, et al. Sentinel surveillance system for early outbreak detection in Madagascar. BMC Public Health. 2010;10:31. doi: 10.1186/1471-2458-10-31 20092624 PMC2823701

[39] Issack MI, Pursem VN, Barkham TMS, Ng L-C, Inoue M, Manraj SS. Reemergence of Dengue in Mauritius. Emerg Infect Dis. 2010;16:716–718. doi: 10.3201/eid1604.091582 20350397 PMC3321960

[40] Gautret P, Simon F, Askling HH, Bouchaud O, Leparc-Goffart I, Ninove L, et al. Dengue type 3 virus infections in European travellers returning from the The Comoros and Zanzibar, February-April 2010. Euro Surveill. 2010;15:19541. doi: 10.2807/ese.15.15.19541-en 20429996

[41] Santé Publique France. Points Epidémiologiques—Dengue—Océan Indien https://www.santepubliquefrance.fr/recherche/#search=dengue&themes=dengue&regions=Oc%C3%A9an%20Indien (accessed September 8, 2021).

[42] Dellagi K, Salez N, Maquart M, Larrieu S, Yssouf A, Silaï R, et al. Serological Evidence of Contrasted Exposure to Arboviral Infections between Islands of the Union of The Comoros (Indian Ocean). PLoS Negl Trop Dis. 2016;10:e0004840. doi: 10.1371/journal.pntd.0004840 27977670 PMC5157944

[43] Larrieu S, Michault A, Polycarpe D, Schooneman F, D’Ortenzio E, Filleul L. data outbreaks: a constant risk for Reunion Island. Results from a seroprevalence study among blood donors. Trans R Soc Trop Med Hyg. 2014;108:57–59. doi: 10.1093/trstmh/trt110 24356127

[44] Vincent M, Larrieu S, Vilain P, Etienne A, Solet J-L, François C, et al. From the threat to the large outbreak: dengue on Reunion Island, 2015 to 2018. Euro Surveill;2019:24. doi: 10.2807/1560-7917.ES.2019.24.47.1900346 31771702 PMC6885751

[45] Sui-Seng S. Institut National de la Statistique et des Etudes Economiques. 853 700 habitants au 1er janvier 2017. INSEE Flash La Réunion-Mayotte. 2019:167.

[46] Besson L. Institut National de la Statistique et des Etudes Economiques. Un Réunionnais sur quatre vit dans les Hauts. INSEE Flash La Réunion-Mayotte. 2017;89.

[47] Marti R, Li Z, Catry T, Roux E, Mangeas M, Handschumacher P, et al. A Mapping Review on Urban Landscape Factors of Dengue Retrieved from Earth Observation Data, GIS Techniques, and Survey Questionnaires. Remote Sens. 2020;12:932. doi: 10.3390/rs12060932

[48] Pascalis H, Biscornet L, Toty C, Hafsia S, Roche M, Desprès P, et al. Complete Genome Sequences of Dengue Virus Type 2 Epidemic Strains from Reunion Island and the Seychelles. Microbiol Resour Announc. 2020;9. doi: 10.1128/MRA.01443-19 31974157 PMC6979306

[49] Boyer S, Foray C, Dehecq J-S. Spatial and Temporal Heterogeneities of Aedes albopictus Density in La Reunion Island: Rise and Weakness of Entomological Indices. PLoS ONE. 2014;9:e91170. doi: 10.1371/journal.pone.0091170 24637507 PMC3956670

[50] Guo X-X, Zhu X-J, Li C-X, Dong Y-D, Zhang Y-M, Xing D, et al. Vector competence of Aedes albopictus and Aedes aegypti (Diptera: Culicidae) for DEN2-43 and New Guinea C virus strains of dengue 2 virus. Acta Trop. 2013;128:566–570. doi: 10.1016/j.actatropica.2013.08.006 23962388

[51] Boussès P, Dehecq JS, Brengues C, Fontenille D. Inventaire actualisé des moustiques (Diptera: Culicidae) de l’île de La Réunion. Océan Indien. Bull Société Pathol Exot. 2013;106:113–125. doi: 10.1007/s13149-013-0288-7 23681758

[52] Tran A, Mangeas M, Demarchi M, Roux E, Degenne P, Haramboure M, et al. Complementarity of empirical and process-based approaches to modelling mosquito population dynamics with Aedes albopictus as an example—Application to the development of an operational mapping tool of vector populations. PLoS ONE. 2020;15:e0227407. doi: 10.1371/journal.pone.0227407 31951601 PMC6968851

[53] Delatte H, Gimonneau G, Triboire A, Fontenille D. Influence of Temperature on Immature Development, Survival, Longevity, Fecundity, and Gonotrophic Cycles of Aedes albopictus, Vector of Chikungunya and Dengue in the Indian Ocean. J Med Entomol. 2009;46:33–41. doi: 10.1603/033.046.0105 19198515

[54] Dieng H, Rahman GMS, Abu Hassan A, Che Salmah MR, Satho T, Miake F, et al. The effects of simulated rainfall on immature population dynamics of Aedes albopictus and female oviposition. Int J Biometeorol. 2012;56:113–120. doi: 10.1007/s00484-011-0402-0 21267602

[55] Barrera R. Considerations for Disrupting Dengue Virus Transmission; Ecology of Aedes aegypti and Current (Nongenetic) Methods of Control. In: Adelman ZN, editor. Genet. Control Malar. Dengue, Elsevier; 2015, p. 103–24.

[56] Barrera R, Amador M, MacKay AJ. Population Dynamics of Aedes aegypti and Dengue as Influenced by Weather and Human Behavior in San Juan. Puerto Rico. PLoS Negl Trop Dis. 2011;5:e1378. doi: 10.1371/journal.pntd.0001378 22206021 PMC3243685

[57] Thomas SJ, Yoon I-K. A review of Dengvaxia: Development to deployment. Hum Vaccines Immunother. 2019;15:2295–2314. doi: 10.1080/21645515.2019.1658503 31589551 PMC6816420

[58] Dehecq J-S, Baville M, Margueron T, Mussard R, Filleul L. La réémergence du chikungunya à La Réunion en 2010: évolution des actions de lutte antivectorielle. Bull Société Pathol Exot. 2011;104:153–160. doi: 10.1007/s13149-010-0121-5 21181327

[59] Achee NL, Grieco JP, Vatandoost H, Seixas G, Pinto J, Ching-NG L, et al. Alternative strategies for mosquito-borne arbovirus control. PLoS Negl Trop Dis. 2019;13:e0006822. doi: 10.1371/journal.pntd.0006822 30605475 PMC6317787

[60] Faraji A, Unlu I. The Eye of the Tiger, the Thrill of the Fight: Effective Larval and Adult Control Measures Against the Asian Tiger Mosquito, Aedes albopictus (Diptera: Culicidae), in North America. J Med Entomol. 2016;53:1029–1047. doi: 10.1093/jme/tjw096 27354440

[61] Flores HA, O’Neill SL. Controlling vector-borne diseases by releasing modified mosquitoes. Nat Rev Microbiol. 2018;16:508–518. doi: 10.1038/s41579-018-0025-0 29777177 PMC7612058

[62] Dunn DW, Follett PA. The sterile insect technique (SIT)-an introduction. Entomol Exp Appl. 2017;164:151–154. 10.1111/eea.12575 PMC5697603 29200471

[63] Proverbs MD. Induced Sterilization and Control of Insects. Annu Rev Entomol. 1969;14:81–102. doi: 10.1146/annurev.en.14.010169.000501 4882184

[64] Bellini R, Calvitti M, Medici A, Carrieri M, Celli G, Maini S. Use of the Sterile Insect Technique Against Aedes albopictus in Italy: First Results of a Pilot Trial. In: Vreysen MJB, Robinson AS, Hendrichs J, editors. Area-Wide Control Insect Pests, Dordrecht: Springer Netherlands; 2007, p. 505–15.

[65] Bellini R, Balestrino F, Medici A, Gentile G, Veronesi R, Carrieri M. Mating Competitiveness of *Aedes albopictus* Radio-Sterilized Males in Large Enclosures Exposed to Natural Conditions. J Med Entomol. 2013;50:94–102. doi: 10.1603/me11058 23427657

[66] Gouagna LC, Damiens D, Oliva CF, Boyer S, Le Goff G, Brengues C, et al. Strategic Approach, Advances, and Challenges in the Development and Application of the SIT for Area-Wide Control of Aedes albopictus Mosquitoes in Reunion Island. Insects. 2020;11:770. doi: 10.3390/insects11110770 33171885 PMC7695178

[67] Yen JH, Barr AR. New hypothesis of the cause of cytoplasmic incompatibility in Culex pipiens L. Nature. 1971;232:657–658. doi: 10.1038/232657a0 4937405

[68] Mains JW, Brelsfoard CL, Rose RI, Dobson SL. Female Adult Aedes albopictus Suppression by Wolbachia -Infected Male Mosquitoes. Nat Sci Rep. 2016;6:33846. doi: 10.1038/srep33846 27659038 PMC5034338

[69] Lebon C, Benlali A, Atyame C, Mavingui P, Tortosa P. Construction of a genetic sexing strain for Aedes albopictus: a promising tool for the development of sterilizing insect control strategies targeting the tiger mosquito. Parasit Vectors. 2018;11:658. doi: 10.1186/s13071-018-3212-y 30583741 PMC6304753

[70] World Health Organization, Special Programme for Research and Training in Tropical Diseases. Dengue Guidelines for Diagnosis, Treatment, Prevention and Control. WHO and TDR. Geneva: 2009.

[71] Atyame CM, Labbé P, Lebon C, Weill M, Moretti R, Marini F, et al. Comparison of Irradiation and Wolbachia Based Approaches for Sterile-Male Strategies Targeting Aedes albopictus. PLoS ONE. 2016;11:e0146834. doi: 10.1371/journal.pone.0146834 26765951 PMC4713058

[72] Zheng X, Zhang D, Li Y, Yang C, Wu Y, Liang X, et al. Incompatible and sterile insect techniques combined eliminate mosquitoes. Nature. 2019;572:56–61. doi: 10.1038/s41586-019-1407-9 31316207

[73] Kittayapong P, Ninphanomchai S, Limohpasmanee W, Chansang C, Chansang U, Mongkalangoon P. Combined sterile insect technique and incompatible insect technique: The first proof-of-concept to suppress Aedes aegypti vector populations in semi-rural settings in Thailand. PLoS Negl Trop Dis. 2019;13:e0007771. doi: 10.1371/journal.pntd.0007771 31658265 PMC6837763

[74] Toledo J, George L, Martinez E, Lazaro A, Han WW, Coelho GE, et al. Relevance of Non-communicable Comorbidities for the Development of the Severe Forms of Dengue: A Systematic Literature Review. PLoS Negl Trop Dis. 2016;10:e0004284. doi: 10.1371/journal.pntd.0004284 26727113 PMC4699776

